# In-hospital thromboprophylaxis variation and the risk of venous thromboembolism after lung cancer surgery: a nationwide cohort study

**DOI:** 10.1093/icvts/ivae081

**Published:** 2024-05-03

**Authors:** Thomas Decker Christensen, Anne Gulbech Ording, Flemming Skjøth, Amalie Lambert Mørkved, Erik Jakobsen, Peter Meldgaard, Rene Horsleben Petersen, Mette Søgaard

**Affiliations:** Department of Cardiothoracic and Vascular Surgery, Aarhus University Hospital, Aarhus, Denmark; Department of Clinical Medicine, Aarhus University, Aarhus, Denmark; Danish Center for Health Services Research, Department of Clinical Medicine, Aalborg University & Aalborg University Hospital, Aalborg, Denmark; Department of Data, Research, and Innovation, Hospital Lillebaelt, University Hospitals of Southern Denmark, Vejle, Denmark; Department of Cardiothoracic and Vascular Surgery, Aarhus University Hospital, Aarhus, Denmark; Department of Clinical Medicine, Aarhus University, Aarhus, Denmark; Department of Thoracic Surgery, Odense University Hospital, Odense, Denmark; Department of Oncology, Aarhus University, Hospital, Aarhus, Denmark; Institute for Clinical Medicine, Faculty of Health Sciences, University of Copenhagen, Copenhagen, Denmark; Department of Cardiothoracic Surgery, Copenhagen University Hospital, Rigshospitalet, Copenhagen, Denmark; Danish Center for Health Services Research, Department of Clinical Medicine, Aalborg University & Aalborg University Hospital, Aalborg, Denmark

**Keywords:** Thromboprophylaxis, Venous thromboembolism, Surgery, Non-small-cell lung cancer, Epidemiology

## Abstract

**OBJECTIVES:**

Venous thromboembolic event (VTE) is a severe complication in patients with lung cancer undergoing thoracic surgery. Nevertheless, because of insufficient evidence, there are no clear guidelines, and VTE prophylaxis practices vary widely. This nationwide cohort study was a comparative study investigating VTE risk in surgical departments that routinely administered in-hospital thromboprophylaxis with low-molecular-weight heparin compared to those that did not.

**METHODS:**

We identified all patients with non-small-cell lung cancer (NSCLC) who underwent surgery in Denmark during 2010–2021. Thoracic surgery was exclusively performed in the 4 university hospitals. Three hospitals implemented in-hospital thromboprophylaxis as standard care since 2000, while the fourth adopted this practice in September 2016. VTE events were assessed at 6-month follow-up according to hospital and study period, using an inverse probability of treatment weighting approach.

**RESULTS:**

We identified 9615 patients. During 6-month follow-up, a total of 190 VTE events were observed, resulting in a weighted rate of 4.5 events per 100 person-years and an absolute risk of 2.2%. There was no clear trend according to hospital site or use of in-hospital thromboprophylaxis with a 2.2% risk in the hospital not using thromboprophylaxis compared to 1.7–3.1% in those that did.

**CONCLUSIONS:**

Use of in-hospital thromboprophylaxis did not affect the risk of VTE after surgery for NSCLC, suggesting that relying solely on in-hospital thromboprophylaxis may be insufficient to mitigate VTE risk in these patients. Further research is warranted to investigate the potential benefits of extended thromboprophylaxis in reducing VTE risk in selected NSCLC surgical patients.

## Graphical Abstract

**Figure:**
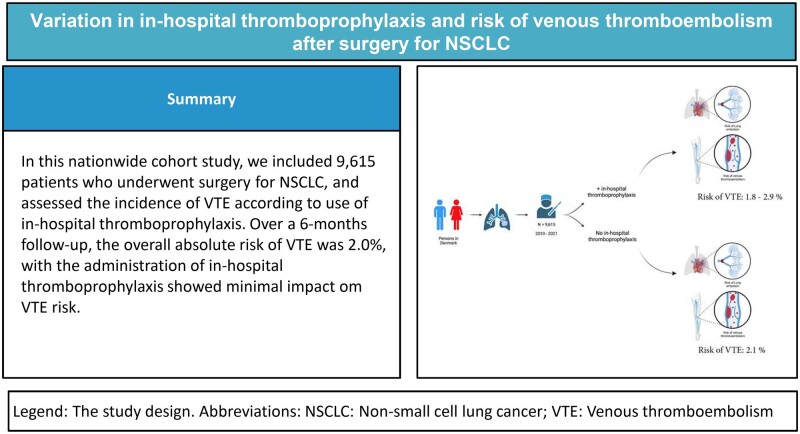

## INTRODUCTION

Venous thromboembolic events (VTEs) represent a major complication following thoracic oncologic surgery associated with significant morbidity and mortality [1]. Current guidelines recommend in-hospital VTE thromboprophylaxis with low-molecular-weight heparin (LMWH) [2]. However, these guidelines are based on weak evidence and lack specific recommendations regarding the duration of thromboprophylaxis and the role of extended prophylaxis after discharge, resulting in substantial practice variation among healthcare centres and physicians [3].

More than one-third of VTEs in patients undergoing surgery for lung cancer are diagnosed after discharge [1, 4–7]. Given the heightened risk of VTE post-discharge and its significant impact on patient morbidity and survival [1], there is a need to reevaluate the adequacy of in-hospital prophylaxis and consider the potential need for extended phrophylaxis after discharge for selected high-risk patients. Instead of a ‘one size fits all’ model relying solely on in-hospital thromboprophylaxis for all, a more personalized and risk-adjusted approach to thromboprophylaxis is warranted. Currently, recommendations for post-discharge prophylaxis are sparse and with low level of evidence for lung cancer patients in contrast to high-risk abdominopelvic and orthopaedic surgeries where evidence supports long-term prophylaxis in the first month following surgery [2].

In Denmark, all thoracic surgical procedures are exclusively performed in 4 University Hospitals. Three of these hospitals have used in-hospital thromboprophylaxis with LMWH as standard of care since 2000, while the fourth adopted this practice in September 2016.

This nationwide cohort study examined whether the risk of VTE was lower in hospitals that routinely administered in-hospital thromboprophylaxis with LMWH compared to those that did not.

## PATIENTS AND METHODS

This was a Danish nationwide cohort study including all patients who underwent a first course of surgery for non-small-cell lung cancer (NSCLC) regardless of stage between 2010 and 2021.

### Setting and data sources

Healthcare in Denmark is provided through a national tax-funded system. All data on diagnoses, procedures and prescription claims are collected in extensive national registries including all patients. A unique personal identifier is assigned to each Danish citizen and all residents upon immigration. This identifier enables linkage of all Danish registries with data on a given individual. Mandatory reporting of new primary malignancies further enhances the availability of cancer-related data in Denmark. For this study, we identified all patients with primary lung cancer through the Danish Lung Cancer Registry (DLCR) [8]. The DLCR is a nationwide clinical quality database that tracks all patients with primary lung cancer in Denmark since 2003. It contains data on, e.g. age, sex, tumour stage, lung function, performance status and initial treatment. By linking records from the DLCR with the Danish National Patient Registry (DNPR) [9], we accessed data on hospitalization and discharge dates, outpatient clinic visits, surgical procedures and discharge diagnoses. Information on antithrombotic treatments and co-medications were retrieved from the Danish National Prescription Registry [10], which has held information on the purchase date and Anatomical Therapeutic Chemical classification code. Information on sex, date of birth, vital status and emigration status was retrieved from the Danish Civil Registration System [11]. All events were tracked and identified through the use of these clinical databases.

### Ethics

The study was approved by the Danish Data Protection Agency through institutional registration (ref. 2019-65). Registry studies do not require ethical approval in Denmark.

### Study population and venous thromboembolic event prophylaxis

The study population included eligible patients aged ≥18 years recorded in the DLCR, who underwent surgical treatment for NSCLC between 2010 and 2021. Exclusion criteria included inconsistent information on date of death, death on the date of surgery, immigration within the year prior to cancer diagnosis, non-NSCLC lung cancer, missing cancer stage data, the prior VTE diagnosis and use of oral anticoagulants within 90 days before surgery. To ensure that baseline data in the DLCR related to the current course of surgery, we further excluded patients with prolonged cancer investigation time (>120 days from registry entry to surgery) and surgery >60 days before registry entry [indicating exploratory thoracotomy (unresectable)], as done previously [12].

The 4 hospitals performing thoracic surgery in Denmark are Aalborg University Hospital (AAUH), Aarhus University Hospital (AUH), Rigshospitalet, Copenhagen University Hospital (RH) and Odense University Hospital (OUH). In 2000, AAUH, AUH and RH implemented in-hospital thromboprophylaxis with LMWH, while OUH adopted this practice in September 2016. Across all sites, Dalteparin (Fragmin) 5000 IE is predominantly used as thromboprophylaxis given subcutaneously once daily. Notable thromboprophylaxis is not continued after discharge (i.e. after 3–7 days of hospitalization).

### Covariates

Data on patient demographics and risk factors comprised age, sex, body mass index, year of cancer diagnosis, cancer stage (pathological tumour-node-metastasis, pathological tumour-node-metastasis stage, respectively), pathology [adenocarcinoma, squamous cell carcinoma or other NSCLs (e.g., large cell carcinoma, carcinoid, adenosquamous carcinoma)], Eastern Cooperative Oncology Group performance status [13], smoking history (reported as pack years) and extent of surgical resection and approach. Missing surgical procedure data in the DLCR were supplemented with surgical procedure codes from the DNPR. Comorbidity assessment involved calculating a modified Charlson Comorbidity Index (CCI) [14], excluding the contribution of cancer diagnoses based on the information obtained from the DNPR. Information on the specific codes used in the study is provided in Supplementary Material, Table S1.

### Outcomes

The primary study outcome was the occurrence of VTE following the surgical procedure, identified through diagnosis codes in the DNRP. Patients were followed for occurrence of VTE for up to 6 months from the surgery date, or until emigration or death, whichever came first. VTE was defined as a composite variable comprising pulmonary embolism, deep venous thrombosis and other VTE types (see Supplementary Material, Table S1 for codes). Both primary and secondary codes from in-patient and ambulatory settings were considered. All-cause mortality was included as a secondary outcome.

### Statistical analyses

Baseline characteristics were summarized descriptively at the time of index surgery, with proportions presented for discrete variables and means and standard deviations for continuous variables and stratified by the hospital department.

We applied time-to-event analyses to analyse outcomes of interest with focus on the differences before and after September 2016, where all hospitals followed the same prophylactic treatment guidelines. To adjust for potential confounding due to regional differences in patient characteristics and hospital preferences in relation to lung cancer treatment, we applied an inverse probability of treatment weighting approach with hospital as exposure. The weights were calculated from logistic regression models with the following potential confounders: calendar period (2010–2012, 2013–2014, 2015–August 2016, September 2016–2018 and 2019–2021), sex, age group (≤49, 50–59, 60–69, 70–79, ≥80 years), indicators for concurrent comorbidity: COPD, hypertension, diabetes, cardiovascular disease and atrial fibrillation, CCI score ≥3, cancer stage, pathology group, days from investigation to intervention and surgical procedure and approach. Categorization of calendar period and age was applied to allow for potential non-linear effect. The average treatment effects weights were stabilized by the marginal hospital distribution. Maximum standardized differences below 0.1 were used as guidelines for successful alignment of baseline characteristics by IPTW (Supplementary Material, Fig. S1). Weighted incidence rates were calculated stratified by hospital department and reported as number of events per 100 person-years over 1-year follow-up. *P*-values of a chi-squared test statistic were calculated to test the hypothesis of equal incidence rate before and after September 2016 [15]. Weighted cumulative incidence functions (by means of the Aalen-Johansen estimator) assuming death as competing risks were used to report VTE risk at 1-year follow-up. Correspondingly, mortality risk was calculated by the Kaplan–Meier estimator.

To exclude the influence of subjects receiving extreme weights, we conducted a sensitivity analysis in which we truncated all weights higher than the 95% percentile. To further refine the weight model, we also performed a sensitivity analysis that incorporated additional data on patient characteristics (e.g. Eastern Cooperative Oncology Group performance status). Due to missing data, this was done by repeating the analyses on a smaller complete dataset.

All analyses were performed with Stata/MP version 17.0 (StataCorp LLC, TX, USA).

## RESULTS

**Figure 1:**
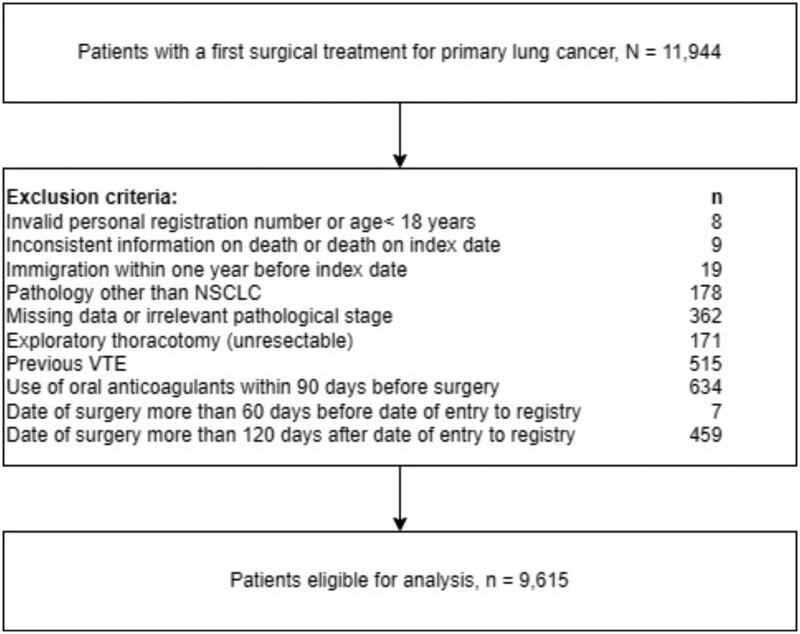
Flowchart of the study population. NSCLC: non-small-cell lung cancer; VTE: venous thromboembolism.

We identified 11 908 patients who underwent a first course of surgical treatment for primary lung cancer in the DLCR between 2010 and 2021. After exclusions, the final study population consisted of 9615 patients with NSCLC who underwent surgery (Fig. 1).

### Baseline characteristics

**Figure 2:**
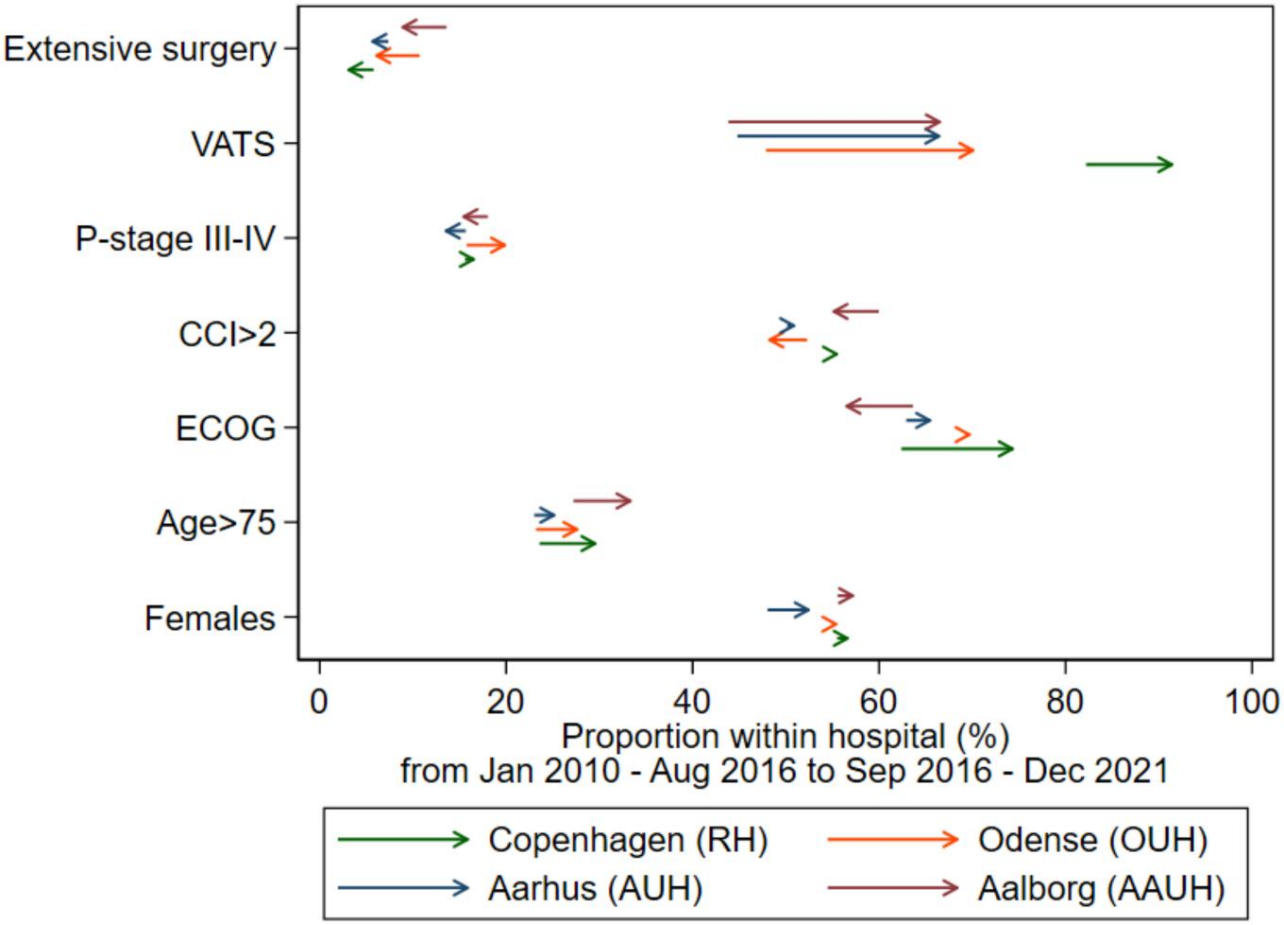
Changes in baseline characteristics over time across hospital sites. The arrows denote the changes from the first period (2010–August 2016) to the second period (September 2016–2021). Extensive surgery is defined as bilobectomy or pneumonectomy. CCI: Charlson Comorbidity Index; ECOG: The Eastern Cooperative Oncology Group performance status; VATS: video-assisted thoracoscopic surgery.

Table 1 displays baseline patient characteristics by hospital site. While most characteristics varied little by site, patients undergoing surgery at AAUH tended to be older with a higher prevalence of comorbidity. Additionally, the number of procedures varied by site, ranging from 1428 at AAUH to 2950 at RH likely reflecting underlying population sizes served by these departments. Across all sites, lobectomy was the primary surgical procedure. The proportion thoracotomy procedures was ∼40% at all centres except RH, where it constituted only 13.4%. Over time, there was a substantial increase in the use of video-assisted thoracoscopic surgery (VATS) across all centres (Fig. 2). Baseline characteristics stratified by observation period within centre are provided in Supplementary Material, Table S2.

**Table 1:** Baseline characteristics of patients undergoing surgery for non-small cell lung cancer in Denmark from 2010 to 2021 by hospital site

	Hospital site			
Characteristics, % (*N*)	Copenhagen (RH) (*N* = 3047)	Odense (OUH) (*N* = 2950)	Aarhus (AUH) (*N* = 2190)	Aalborg (AAUH) (*N* = 1428)
Study period				
January 2010–August 2016	51.6 (1573)	48.4 (1427)	47.8 (1047)	44.3 (632)
September 2016–December 2021	48.4 (1474)	51.6 (1523)	52.2 (1143)	55.7 (796)
Patient characteristics				
Females	56.0 (1707)	55.3 (1631)	50.3 (1102)	56.4 (806)
Mean age at surgery (SD), years	68.1 (9.4)	68.0 (9.3)	68.0 (9.2)	1. (9.6)
<50	3.6 (109)	3.4 (99)	3.0 (66)	1. (43)
50–59	12.7 (388)	13.4 (395)	13.2 (290)	1. (190)
60–69	35.1 (1068)	36.2 (1068)	34.7 (761)	1. (448)
70–79	40.6 (1236)	38.1 (1124)	41.3 (904)	41.5 (592)
>80	8.1 (246)	8.9 (264)	7.7 (169)	10.9 (155)
Body mass index^a^				
<18.5	4.8 (119)	4.2 (110)	4.3 (88)	1. (53)
18.5–24.9	48.5 (1213)	45.3 (1185)	44.4 (914)	1. (593)
25–29.9	31.9 (797)	33.9 (886)	35.1 (721)	1. (401)
30–34.9	11.0 (275)	12.4 (323)	11.8 (243)	13.6 (174)
>35	3.9 (98)	4.2 (110)	4.4 (91)	4.5 (58)
Smoking, pack year^a^				
None	8.8 (240)	6.7 (173)	6.9 (132)	1. (99)
<15	7.2 (195)	6.6 (169)	6.8 (131)	9.2 (117)
>15	84.0 (2288)	86.7 (2238)	86.3 (1658)	83.0 (1055)
ECOG PS^a^				
Fully active	68.2 (1983)	69.5 (1950)	64.2 (1375)	1. (845)
Reduced activity	31.8 (926)	30.5 (857)	35.8 (766)	40.3 (571)
Comorbidity				
CCI score ≥3	55.3 (1684)	50.2 (1481)	50.5 (1106)	57.3 (818)
COPD	22.6 (688)	20.5 (606)	16.4 (360)	20.2 (289)
Hypertension	30.6 (931)	26.3 (777)	22.3 (489)	28.9 (413)
Diabetes mellitus	9.8 (298)	7.9 (233)	7.3 (160)	10.4 (149)
Cardiovascular disease	21.0 (639)	18.4 (542)	20.9 (458)	23.0 (329)
Atrial fibrillation/flutter	5.7 (174)	4.1 (122)	5.4 (119)	5.7 (82)
Antiplatelet therapy at time of surgery	4.0 (123)	4.9 (144)	4.1 (90)	4.8 (68)
Cancer characteristics				
Staging based on pTNM				
I	63.1 (1922)	55.4 (1635)	61.5 (1346)	61.3 (876)
II	20.9 (637)	26.7 (788)	24.0 (525)	22.1 (315)
III	13.7 (417)	15.6 (461)	11.8 (258)	14.5 (207)
IV	2.3 (71)	2.2 (66)	2.8 (61)	2.1 (30)
Pathology				
Adenocarcinoma	57.7 (1759)	59.0 (1741)	55.9 (1225)	57.8 (825)
Squamous cell carcinoma	18.4 (561)	26.2 (773)	23.4 (513)	27.5 (392)
Other NSCLC	23.0 (701)	14.6 (431)	20.3 (444)	14.4 (206)
Missing	0.9 (26)	0.2 (5)	0.4 (8)	0.4 (5)
Surgical characteristics				
Mean time from start of cancer investigation to surgery (SD) (days)	44.5 (17.9)	42.1 (18.1)	42.1 (17.5)	43.8 (16.1)
Surgical procedure				
Sublobar resections	9.9 (303)	12.6 (371)	11.5 (251)	14.8 (211)
Lobectomy	85.6 (2607)	79.1 (2333)	82.1 (1797)	74.2 (1060)
Bilobectomy	2.7 (83)	3.8 (112)	3.3 (73)	5.6 (80)
Pneumonectomy	1.8 (54)	4.5 (134)	3.2 (69)	5.4 (77)
Surgical approach				
Thoracotomy	13.4 (407)	40.7 (1200)	43.9 (962)	43.6 (622)
VATS	86.6 (2640)	59.3 (1750)	56.1 (1228)	56.4 (806)

a Proportion with non-missing data; information on missing data is provided in Supplementary Material, Table S3.

AAUH: Aalborg University Hospital; AUH: Aarhus University Hospital; CCI: Charlson Comorbidity Index; COPD: chronic obstructive pulmonary disease; ECOG PS: The Eastern Cooperative Oncology Group performance status; NSCLC: non-small cell lung cancer; OUH: Odense University Hospital; pTNM: pathological tumour-node-metastasis stage; RH: Rigshospitalet, Copenhagen University Hospital; SD: standard deviation; VATS: video-assisted thoracoscopic surgery.

### Venous thromboembolism

**Figure 3:**
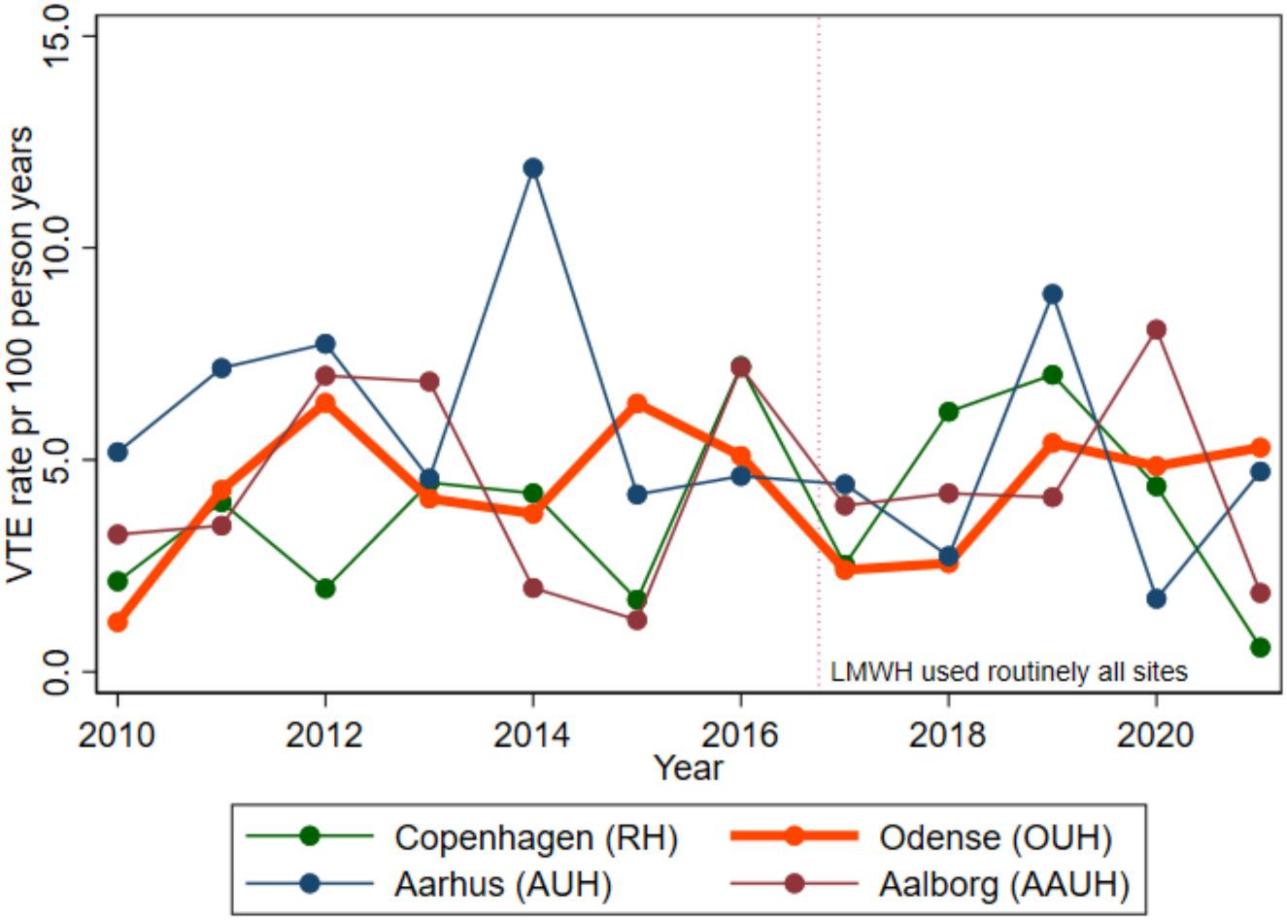
Weighted rates of venous thromboembolism at 6-month follow-up in patients who underwent surgery for primary lung cancer displayed by year and hospital. LMWH: low-molecular-weight heparin; VTE: venous thromboembolism.

Overall, we observed 190 VTE events during 6-month follow-up, corresponding to a weighted rate of 4.5 events per 100 person-years and an absolute risk of 2.2%. The overall risk of VTE remained relatively stable over time. The weighted rates of VTE at each site fluctuated widely by study year, with no clear trend according to hospital site or use of in-hospital thromboprophylaxis (Fig. 3).

**Figure 4:**
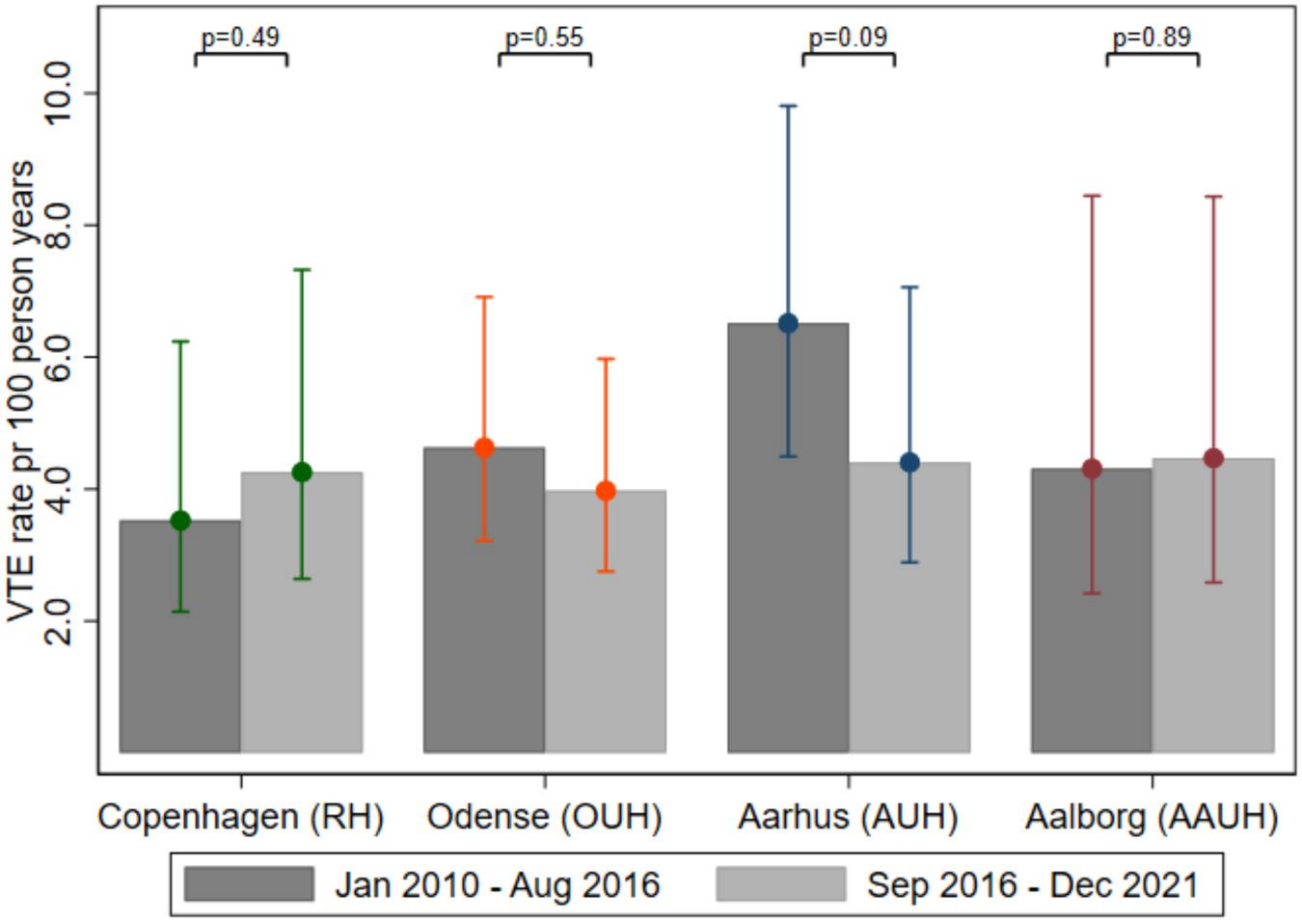
Weighted rates of venous thromboembolism at 6-month follow-up in patients who underwent surgery for primary stratified by hospital and time period. VTE: venous thromboembolism.

Table 2 presents the number of events, weighted rates and weighted absolute risks by period and hospital site. In the hospital not using in-hospital thromboprophylaxis in the first time period, the risk of VTE was 2.2% risk of VTE compared to 1.7%, 2.1% and 3.1%, respectively, in the hospitals using in-hospital prophylaxis. The overall risk of VTE decreased slightly over time (Table 2). Regardless of use of in-hospital thromboprophylaxis, the weighted rate of VTE was not statistically significantly different between January 2010 and August 2016 and September 2016 and December 2021 at any of hospital site (Fig. 4).

**Table 2:** Number of events, weighted rates and absolute risk of venous thromboembolism at 6 months follow-up in patients undergoing surgery for non-small-cell lung cancer stratified by hospital and time period

	January 2010–August 2016			September 2016–December 2021		
Surgical site	Events	Weighted rate	Weighted risk (%)	Events	Weighted rate	Weighted risk (%)
Copenhagen (RH)	23	3.5	1.7	27	4.3	2.0
Odense (OUH)	32	4.6	2.2	28	4.0	1.9
Aarhus (AUH)	30	6.5	3.1	23	4.4	2.1
Aalborg (AAUH)	13	4.3	2.1	14	4.5	2.2
All sites	98	4.8	2.3	92	4.3	2.1

Rates are events divided by person time for 100 years.

AAUH: Aalborg University Hospital; AUH: Aarhus University Hospital; OUH: Odense University Hospital; RH: Rigshospitalet, Copenhagen University Hospital.

### All-cause mortality

A total of 413 deaths occurred during the 6-month follow-up, corresponding to a mortality rate of 8.6 deaths per 100 person-years and an overall absolute risk of 4.2%. Mortality rates decreased from the first to the last study period, with considerable variation between hospital centres (Supplementary Material, Table S4).

### Sensitivity analysis

Sensitivity analyses with truncation of extreme weights and with a more elaborate weighting model had virtually no impact on estimates of VTE and mortality (data not shown).

## DISCUSSION

Our findings indicate that the administration of in-hospital thromboprophylaxis did not affect the risk of VTE at 6 months in patients who underwent surgery for NSCLC. This suggests that relying solely on in-hospital thromboprophylaxis is inadequate for mitigating VTE risk in this patient population. Thus, a research priority should focus on identifying patients at high risk of VTE after discharge for lung cancer surgery where extended prophylaxis may be valuable.

Our findings cohere with a previous systematic review, estimating a mean VTE risk of ∼2.0% over 16 months, although estimates varied widely across studies [4]. A recent Canadian cohort study including 12 626 patients undergoing lung cancer surgery demonstrated a 1-year VTE risk of 2.7% [1]. However, it is important to acknowledge that most observational cohort studies, including ours, likely underestimate the true incidence of VTE. Conversely, intervention studies involving screening for VTE (e.g. using lower extremity ultrasound) have documented higher VTE incidence due to the diagnosis of asymptomatic VTE [16].

Current guidelines from the American Association for Thoracic Surgery and the European Society of Thoracic Surgeons recommend prolonged prophylaxis for 30 days after surgery in patients undergoing extended resections. However, these recommendations are supported by a low level of evidence [2]. Consequently, the optimal length of postoperative thromboprophylaxis in lung cancer patients is unknown. In a previous trial [17], lung cancer patients undergoing VATS lobectomy were randomly assigned to either subcutaneous dalteparin (Fragmin) 5000 IE once daily or no intervention during hospitalization. Coagulation was assessed before, during and 2 days after surgery using standard coagulation blood tests, thromboelastometry (ROTEM) and thrombin generation. The trial found no significant differences between the 2 groups regarding bleeding, thromboembolic events or the coagulation profiles, indicating that once-daily LMWH thromboprophylaxis did not alter the coagulation profile *per se*. While it may be feasible to conduct a randomized controlled trial testing various thromboprophylaxis regimens, including the option of no thromboprophylaxis, during hospitalization for patients undergoing lung cancer surgery, such a trial would require a large sample size to achieve sufficient statistical power.

The findings of the current study indicate that in-hospital thromboprophylaxis may not be as important as previously thought, potentially due in part to the implementation and effectiveness of the ERAS guidelines in thoracic surgery [18]. Previous studies have shown that a substantial proportion of VTE events occurs after discharge [5–7], suggesting that a focus on post-discharge prophylaxis may be equally important. However, prophylactic anticoagulation also has drawbacks, including increased risk of bleeding, costs, patient discomfort and inconvenience. Oral alternatives such as direct-acting oral anticoagulants (DOACs) obviate the need for daily injections with documented comparable efficacy to LMWH [19, 20]. This calls for a need to personalize decision-making about thromboprophylaxis and consider extended prophylaxis for high-risk patients such as those with large resections, advanced cancer stage, central tumours compressing veins or undergoing treatment with systemic therapies (e.g. neo-adjuvant chemotherapy). This approach could have implications for current clinical practice, particularly considering the increasing use of VATS and shorter hospital stays, where patients are mobilized, monitored, and given respiratory physiotherapy very intensely within a few days of admission [21]. Given this, thromboprophylaxis may be more relevant after discharge.

Strengths of this study include data from a nationwide real-world population who had access to free health care without exclusions based on physical fitness or other factors. This approach reduced the potential selection biases observed in previous studies including specific subsets of cancer patients. The data used in this study were collected for quality assurance and administrative purposes, independent of the research question and the DLCR encompasses the entire eligible population, enhancing the generalizability of the findings.

### Limitations

Study limitations include the reliance on complete and accurate coding, although the positive predictive value of the VTE diagnoses in the DNPR is >90% [22]. Despite adherence to national guidelines, variations may exist between the 4 centres in practice such as VATS usage, nursing care, physiotherapy, medication choice and other unaccounted variables within our inverse probability of treatment weighting approach. Consequently, residual confounding likely persists. Moreover, administration of LMWH prophylaxis during hospital stay is not recorded in the registries, necessitating the use of treatment centres as a proxy for prophylaxis administration, introducing potential exposure misclassification. Nevertheless, it is important to note that, as standard practice, all Danish centres followed guidelines by administering LMWH prophylaxis during hospitalization for all patients undergoing surgery, with the exception of OUH before September 2016. Finally, the limited number of VTE events resulted in considerable variability between centres and over time. Data on VTE severity and whether some were incidental findings during follow-up scans, and the specific causes of death were unavailable. Therefore, we were unable to ascertain whether some deaths may have been attributable to VTE.

## CONCLUSIONS

This nationwide cohort study demonstrated that administration of in-hospital thromboprophylaxis did not affect the risk of VTE after surgery for NSCLC. Combined with the growing body of evidence on post-discharge risk, this suggests that in-hospital thromboprophylaxis may not be sufficient to mitigate VTE risk. Further research is warranted to investigate the potential clinical benefits of extended thromboprophylaxis in reducing VTE risk in NSCLC surgical patients.

## Supplementary Material

ivae081_Supplementary_Data

## Data Availability

The data were provided by the Danish Health Data Authority, The Danish Clinical Quality Program—National Clinical Registries (RKKP) and The DLCR. Owing to data protection rules, we are not allowed to share individual-level data. Other researchers who fulfil the requirements set by the data providers could obtain similar data.

## ABBREVIATIONS

AAUH: Aalborg University Hospital
AUH: Aarhus University Hospital
DLCR: Danish Lung Cancer Registry
DNPR: Danish National Patient Registry
DOACs: Direct-acting oral anticoagulants
LMWH: Low-molecular-weight heparin
NSCLC: Non-small-cell lung cancer
OUH: Odense University Hospital
RH: Rigshospitalet, Copenhagen University Hospital
VATS: Video-assisted thoracoscopic surgery
VTE: Venous thromboembolic event
