# Supplementary material for: In-hospital thromboprophylaxis variation and the risk of venous thromboembolism after lung cancer surgery: a nationwide cohort study
Source: Interdiscip Cardiovasc Thorac Surg. 2024 May 3;38(5):ivae081. doi: 10.1093/icvts/ivae081 (PMC11090992; doi:10.1093/icvts/ivae081)

**Supplemental Table 1:** ICD and ATC codes used in the study.

|  | **International Classification of Diseases 10th revision (ICD-10) code** | **International Classification of Diseases 8th revision (ICD-8) code** | **Anatomical Therapeutic Chemical (ATC) code** | **SKS code** |
| --- | --- | --- | --- | --- |
| **Venous thromboembolism** |  |  |  |  |
| VTE overall | I26 I636 I676 I801 I802 I803 I808 I809 I81 I822 I823 I828 I829 H348E H348F T817C |  |  |  |
| **Comorbidity** |  |  |  |  |
| COPD | J44 |  |  |  |
| Hypertension | I10 I11 I12 I13 I15 | 400 401 402 403 404 |  |  |
| Diabetes mellitus | E10 E11 E12 E13 E14 H360 | 249 250 |  |  |
| Cardiovascular disease | I48 I20 I21 I23 I24 I25 I46 | 42793 42794 41009 1099 41109 41199 41209 1299 41309 41399 41409 1499 42727 |  |  |
| Atrial fibrillation | I48 |  |  |  |
| Charlson Comorbidity Index | I21 I22 I23 I50 I110 I130 I132 I70 I71 I72 I73 I74 I77 I60 I61 I62 I63 I64 I65 I66 I67 I68 I69 G45 G46 F00 F01 F02 F03 F051 G30 J40 J41 J42 J43 J44 J45 J46 J47 J60 J61 J62 J63 J64 J65 J66 J67 J684 J701 J703 J841 J920 J961 J982 J983 M05 M06 M08 M09 M30 M31 M32 M33 M34 M35 M36 D86 K221 K25 K26 K27 K28 B18 K700 K701 K702 K703 K709 K71 K73 K74 K760 E100 E101 E109 E110 E111 E119 G81 G82 I12 I13 N00 N01 N02 N03 N04 N05 N07 N11 N14 N17 N18 N19 Q61 E102 E103 E104 E105 E106 E107 E108 E112 E113 E114 E115 E116 E117 E118 B150 B160 B162 B190 K704 K72 K766 I85 B21 B22 B23 B24 | 410 42709 42710 42711 42719 42899 78249 440 441 442 443 444 445 430 431 432 433 434 435 436 437 438 29009 29010 29011 29012 29013 29014 29015 29016 29017 29018 29019 29309 490 491 492 493 515 516 517 518 712 716 734 446 13599 53091 53098 531 532 533 534 571 57301 57304 249 250 344 403 404 580 581 582 583 584 59009 59319 7531 792 24901 24902 24903 24904 24905 24908 25001 25002 25003 25004 25005 25008 07000 07002 07004 07006 07008 57300 4560  07983 |  |  |
| **Medication** |  |  |  |  |
| Apixaban |  |  | B01AF02 |  |
| Aspirin |  |  | B01AC06 |  |
| Clopidogrel |  |  | B01AC04 |  |
| Dabigatran |  |  | B01AE07 |  |
| Edoxaban |  |  | B01AF03 |  |
| Persantin |  |  | B01AC07 |  |
| Phenprocoumon |  |  | B01AA04 |  |
| Prasugrel |  |  | B01AC22 |  |
| Rivaroxaban |  |  | B01AF01 |  |
| Ticagrelor |  |  | B01AC24 |  |
| Warfarin |  |  | B01AA03 |  |
| **Surgical procedure codes** |  |  |  |  |
| Wedge resection |  |  |  | KGDB10 |
| Thoracoscopic wedge resection |  |  |  | KGDB11 |
| Segment resection |  |  |  | KGDB20 |
| Thoracoscopic segment resection |  |  |  | KGDB21 |
| Lobectomy |  |  |  | KGDC00 |
| Thoracoscopic lobectomy |  |  |  | KGDC01 |
| Other open lobectomy |  |  |  | KGDC96 |
| Other thoracoscopic lobectomy |  |  |  | KGDC97 |
| Bilobectomy |  |  |  | KGDC10 |
| Thoracoscopic bilobectomy |  |  |  | KGDC11 |
| Extendended lobectomy or bilobectomy |  |  |  | KGDC13 |
| Lobectomy with sleeve resection |  |  |  | KGDC20 |
| Lobectomy and other lung resection |  |  |  | KGDC26 |
| Lobectomy and segment resection |  |  |  | KGDC23 |
| Pneumonectomy |  |  |  | KGDD00 |
| Thoracoscopic pneumonectomy |  |  |  | KGDD01 |
| Other pneumonectomy |  |  |  | KGDD96 |
| Other thoracoscopic pneumonectomy |  |  |  | KGDD97 |
| Pleuropneumonectomy |  |  |  | KGDD10 |
| Thoracoscopic pleuropneumonectomy |  |  |  | KGDD11 |
| Extended pneumonectomy |  |  |  | KGDD20 |
| Pneumonectomy and resection of bronchus or trachea |  |  |  | KGDD26 |
| Pneumonectomy and resection of carina |  |  |  | KGDD23 |
| Thoracoscopy |  |  |  | KGAA31 |
| Other lung resection |  |  |  | KGDB96 |
| Other thoracoscopic lung resection |  |  |  | KGDB97 |
| Excision of pathologic lung tissue |  |  |  | KGDA20 |
| Thoracoscopic excision of pathologic lung tissue |  |  |  | KGDA21 |
| Other biopsy, incision, excision, revision, or suture on lung |  |  |  | KGDA96 |
| Other thoracoscopic biopsy, incision, excision, revision, or suture on lung |  |  |  | KGDA97 |
| Other bronchoscopic incision, excision, revision or suture on lung |  |  |  | KGDA98 |
| Resection of the thoracic wall |  |  |  | KGAE16 |
| Anterior or posterolateral thoracotomy |  |  |  | KGAB10 |
| Transaxillary thoracotomy |  |  |  | KGAB13 |
| Mini-thoracotomy |  |  |  | KGAB96A |
| Median sternotomy |  |  |  | KGAB20 |

Abbreviations: VTE, venous thromboembolism; DVT, deep venous thrombosis; PE, pulmonary embolism

**Supplemental Table 2:** Baseline characteristics of patients undergoing surgery for non-small cell lung cancer in Denmark in 2010-2021 by hospital site.

|  | Hospital site | | | | | | | |
| --- | --- | --- | --- | --- | --- | --- | --- | --- |
| Characteristics, % (N) | Copenhagen (RH)  (N=3,047) | | Odense (OUH)  (N=2,950) | | Aarhus (AUH)  (N=2,190) | | Aalborg (AAUH)  (N=1,428) | |
| Study period | 2010-8/2016 | 9/2016-2021 | 2010-8/2016 | 9/2016-2021 | 2010-8/2016 | 9/2016-2021 | 2010-8/2016 | 9/2016-2021 |
| N | 1,573 | 1,474 | 1427 | 1523 | 1047 | 1143 | 632 | 796 |
| Patient characteristics |  |  |  |  |  |  |  |  |
| Females | 55.5 (873) | 56.6 (834) | 55.2 (788) | 55.4 (843) | 48.0 (503) | 52.4 (599) | 55.5 (351) | 57.2 (455) |
| Mean age at surgery (SD), years | 67.3 (9.5) | 68.9 (9.1) | 67.6 (9.5) | 68.5 (9.1) | 67.4 (9.3) | 68.5 (9.0) | 67.8 (9.9) | 69.6 (9.2) |
| - <50 | 4.3 (67) | 2.8 (42) | 4.1 (59) | 2.6 (40) | 3.4 (36) | 2.6 (30) | 4.0 (25) | 2.3 (18) |
| - 50-59 | 13.4 (210) | 12.1 (178) | 13.4 (191) | 13.4 (204) | 15.1 (158) | 11.5 (132) | 15.5 (98) | 11.6 (92) |
| - 60-69 | 38.5 (605) | 31.4 (463) | 37.6 (536) | 34.9 (532) | 36.2 (379) | 33.4 (382) | 32.0 (202) | 30.9 (246) |
| - 70-79 | 36.6 (576) | 44.8 (660) | 36.1 (515) | 40.0 (609) | 37.1 (388) | 45.1 (516) | 38.8 (245) | 43.6 (347) |
| >80 | 7.3 (115) | 8.9 (131) | 8.8 (126) | 9.1 (138) | 8.2 (86) | 7.3 (83) | 9.8 (62) | 11.7 (93) |
| Body mass index* |  |  |  |  |  |  |  |  |
| - <18.5 | 5.1 (59) | 4.4 (60) | 4.2 (51) | 4.2 (59) | 3.9 (38) | 4.6 (50) | 4.9 (28) | 3.6 (25) |
| - 18.5-24.9 | 51.3 (592) | 46.0 (621) | 48.1 (583) | 43.0 (602) | 49.9 (484) | 39.6 (430) | 47.7 (275) | 45.3 (318) |
| - 25-29.9 | 30.4 (350) | 33.1 (447) | 33.7 (409) | 34.0 (477) | 33.4 (324) | 36.5 (397) | 30.3 (175) | 32.2 (226) |
| - 30-34.9 | 9.7 (112) | 12.1 (163) | 10.8 (131) | 13.7 (192) | 9.2 (89) | 14.2 (154) | 13.7 (79) | 13.5 (95) |
| >35 | 3.5 (40) | 4.3 (58) | 3.2 (39) | 5.1 (71) | 3.6 (35) | 5.2 (56) | 3.5 (20) | 5.4 (38) |
| Smoking, pack year* |  |  |  |  |  |  |  |  |
| None | 9.5 (132) | 8.1 (108) | 6.1 (74) | 7.3 (99) | 6.1 (56) | 7.6 (76) | 8.0 (46) | 7.6 (53) |
| - <15 | 5.9 (82) | 8.5 (113) | 6.3 (77) | 6.8 (92) | 6.5 (60) | 7.1 (71) | 8.7 (50) | 9.6 (67) |
| >15 | 84.6 (1180) | 83.4 (1108) | 87.6 (1068) | 86.0 (1170) | 87.4 (807) | 85.3 (851) | 83.2 (476) | 82.8 (579) |
| ECOG PS* |  |  |  |  |  |  |  |  |
| - Fully active | 62.4 (934) | 74.3 (1049) | 69.3 (931) | 69.7 (1019) | 62.9 (641) | 65.4 (734) | 63.6 (399) | 56.5 (446) |
| - Reduced activity | 37.6 (563) | 25.7 (363) | 30.7 (413) | 30.3 (444) | 37.1 (378) | 34.6 (388) | 36.4 (228) | 43.5 (343) |
| Comorbidity |  |  |  |  |  |  |  |  |
| CCI score ≥3 | 55.1 (867) | 55.4 (817) | 52.3 (746) | 48.3 (735) | 50.1 (525) | 50.8 (581) | 60.0 (379) | 55.2 (439) |
| COPD | 18.1 (285) | 27.3 (403) | 15.8 (226) | 25.0 (380) | 13.9 (146) | 18.7 (214) | 20.6 (130) | 20.0 (159) |
| Hypertension | 29.4 (462) | 31.8 (469) | 27.3 (389) | 25.5 (388) | 23.9 (250) | 20.9 (239) | 30.5 (193) | 27.6 (220) |
| Diabetes mellitus | 9.2 (145) | 10.4 (153) | 7.7 (110) | 8.1 (123) | 7.7 (81) | 6.9 (79) | 10.6 (67) | 10.3 (82) |
| Cardiovascular disease | 22.9 (360) | 18.9 (279) | 20.3 (290) | 16.5 (252) | 21.9 (229) | 20.0 (229) | 24.7 (156) | 21.7 (173) |
| Atrial fibrillation/flutter | 6.7 (105) | 4.7 (69) | 4.6 (65) | 3.7 (57) | 6.6 (69) | 4.4 (50) | 6.8 (43) | 4.9 (39) |
| Antiplatelet therapy at time of surgery | 4.3 (67) | 3.8 (56) | 5.5 (79) | 4.3 (65) | 4.1 (43) | 4.1 (47) | 4.1 (26) | 5.3 (42) |
| Cancer characteristics |  |  |  |  |  |  |  |  |
| Staging based on pTNM |  |  |  |  |  |  |  |  |
| I | 62.1 (977) | 64.1 (945) | 58.4 (834) | 52.6 (801) | 59.5 (623) | 63.3 (723) | 56.8 (359) | 64.9 (517) |
| II | 22.3 (351) | 19.4 (286) | 25.8 (368) | 27.6 (420) | 24.8 (260) | 23.2 (265) | 25.2 (159) | 19.6 (156) |
| III | 12.9 (203) | 14.5 (214) | 13.2 (189) | 17.9 (272) | 11.7 (123) | 11.8 (135) | 14.9 (94) | 14.2 (113) |
| IV | 2.7 (42) | 2.0 (29) | 2.5 (36) | 2.0 (30) | 3.9 (41) | 1.7 (20) | 3.2 (20) | 1.3 (10) |
| Pathology |  |  |  |  |  |  |  |  |
| Adenocarcinoma | 49.1 (773) | 66.9 (986) | 55.1 (786) | 62.7 (955) | 47.3 (495) | 63.9 (730) | 52.5 (332) | 61.9 (493) |
| Squamous cell carcinoma | 19.1 (300) | 17.7 (261) | 28.7 (410) | 23.8 (363) | 26.2 (274) | 20.9 (239) | 28.2 (178) | 26.9 (214) |
| Other NSCLC or missing | 31.8 (500) | 15.4 (227) | 16.2 (231) | 13.5 (205) | 26.6 (278) | 15.2 (174) | 19.3 (122) | 11.2 (88) |
| Surgical characteristics |  |  |  |  |  |  |  |  |
| Mean time from start of cancer investigation to surgery (SD), days | 45.0 (17.5) | 44.1 (18.3) | 42.8 (18.6) | 41.4 (17.7) | 40.9 (17.6) | 43.2 (17.4) | 41.2 (14.7) | 45.9 (16.8) |
| Surgical procedure |  |  |  |  |  |  |  |  |
| Sublobar resections | 10.9 (171) | 9.0 (132) | 14.7 (210) | 10.6 (161) | 12.6 (132) | 10.4 (119) | 14.2 (90) | 15.2 (121) |
| Lobectomy | 83.3 (1311) | 87.9 (1296) | 74.6 (1064) | 83.3 (1269) | 80.0 (838) | 83.9 (959) | 72.2 (456) | 75.9 (604) |
| Bilobectomy | 3.1 (48) | 2.4 (35) | 4.7 (67) | 3.0 (45) | 3.9 (41) | 2.8 (32) | 6.0 (38) | 5.3 (42) |
| Pneumonectomy | 2.7 (43) | 0.7 (11) | 6.0 (86) | 3.2 (48) | 3.4 (36) | 2.9 (33) | 7.6 (48) | 3.6 (29) |
| Surgical approach |  |  |  |  |  |  |  |  |
| Thoracocomy | 17.8 (280) | 8.6 (127) | 52.1 (744) | 29.9 (456) | 55.2 (578) | 33.6 (384) | 56.2 (355) | 33.5 (267) |
| VATS | 82.2 (1293) | 91.4 (1347) | 47.9 (683) | 70.1 (1067) | 44.8 (469) | 66.4 (759) | 43.8 (277) | 66.5 (529) |

*Proportion with non-missing data; information on missing data is provided in Supplementary

Abbreviations: AAUH, Aalborg University Hospital; AUH, Aarhus University Hospital; ECOG PS, The Eastern Cooperative Oncology Group performance status; CCI, Charlson Comorbidity Index; COPD, Chronic obstructive pulmonary disease; pTNM, pathological tumor-node-metastasis stage; NSCLC, Non-small cell lung cancer; OUH, Odense University Hospital; RH, Rigshospitalet, Copenhagen University Hospital; VATS, Video-assisted thoracoscopic surgery

**Supplementary Table 3:** Information on missing values according to hospital site

|  | **Hospital site** | | | |
| --- | --- | --- | --- | --- |
| **Variable, % (N)** | **Copenhagen (RH)**  **(N=3,047)** | **Odense (OUH)**  **(N=2,950)** | **Aarhus (AUH)**  **(N=2,190)** | **Aalborg (AAUH)**  **(N=1,428)** |
| Body mass index | 17.9 (545) | 11.4 (336) | 6.1 (133) | 10.4 (149) |
| Smoking | 10.6 (324) | 12.5 (370) | 12.3 (269) | 11.0 (157) |
| ECOG PS | 4.5 (138) | 4.8 (143) | 2.2 (49) | 0.8 (12) |

Abbreviations: ECOG PS, The Eastern Cooperative Oncology Group performance status

**Supplementary Table 4:** Number of deaths, weighted rate, and absolute risk of all-cause mortality six months follow-up in patients undergoing surgery for non-small cell lung cancer stratified by hospital and time period.

|  | **Jan 2010 - Aug 2016** | | |  | **Sep 2016 – Dec 2021** | | |
| --- | --- | --- | --- | --- | --- | --- | --- |
| **Surgical site** | **Events** | **Weighted rate** | **Weighted risk,**  **% (95% CI)** |  | **Events** | **Weighted rate** | **Weighted risk,**  **% (95% CI)** |
| Copenhagen | 83 | 10.4 | 5.1 |  | 40 | 7.6 | 3.6 |
| Odense | 77 | 10.0 | 4.9 |  | 60 | 7.5 | 3.7 |
| Aarhus | 59 | 10.7 | 5.2 |  | 29 | 5.9 | 2.9 |
| Aalborg | 33 | 9.7 | 4.7 |  | 32 | 7.3 | 3.5 |
| All sites | 252 | 19.2 | 5.0 |  | 161 | 7.1 | 3.4 |

Rates are events divided by person time pr 100 years.

Supplementary Figure 1: Unweighted and weighted maximum standardized differences between hospital sites of baseline characteristics, weighted differences below 0.1 are considered as indication for balance between sites.


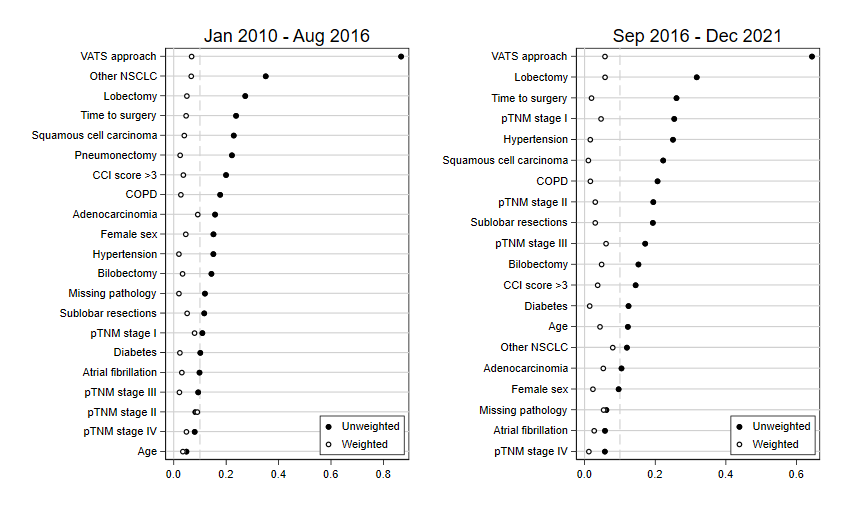

Supplement: ivae081_Supplementary_Data [file ivae081_supplementary_data.docx]
